# *Npr2* mutant mice show vasodilation and undeveloped adipocytes in mesentery

**DOI:** 10.1186/s13104-021-05853-9

**Published:** 2021-11-27

**Authors:** Chizuru Sogawa-Fujiwara, Yasuhiro Fujiwara, Atsuki Hanagata, Qunhui Yang, Taiki Mihara, Noriyuki Kaji, Tetsuo Kunieda, Masatoshi Hori

**Affiliations:** 1 Veterinary Pharmacology, Graduate School of Agriculture and Life Sciences, The University of Tokyo, 1-1-1 Yayoi, Bunkyo-ku, Tokyo, 113-8657 Japan; 2 Laboratory of Pathology and Development, Institute for Quantitative Biosciences, The University of Tokyo, 1-1-1 Yayoi, Bunkyo-ku, Tokyo, 113-0032 Japan; 3 Laboratory of Veterinary Pharmacology, School of Veterinary Medicine, Azabu University, 1-17-71 Fuchinobe, Chuo-ku, Sagamihara, Kanagawa 252-5201 Japan; 4 Faculty of Veterinary Medcine, Okayama University of Science, 1-3 Ikoino-oka, Imabari, Ehime 794-8555 Japan

**Keywords:** CNP, NPR-B, Blood vessel, Adipocyte, Intestinal disorder

## Abstract

**Objective:**

The biological importance for the signaling of C-type natriuretic peptide (CNP) and natriuretic peptide receptor B (NPR-B) has been recognized. However, the details remain unclear and are debatable. The *Npr2* is a gene of NPR-B, and we previously reported a unique phenotype of a spontaneous mutant mouse lacking *Npr2* (*Npr2*^*slw/slw*^), such as severe ileus-like disorder with bloodless blood vessels. In this study, we analyzed the bloodless mesenteric vascular morphology of *Npr2*^*slw/slw*^ by histological observation to clarify the effects of the CNP/NPR-B signal deficiency.

**Results:**

Blood vessels in the mesentery were clearly dilated in the preweaning *Npr2*^*slw/slw*^ mice. Additionally, in the *Npr2*^*slw/slw*^ mice, the lacteals were partially dilation or randomly direction mucosal epithelial cells in villi, and mesenteric adipocytes were undeveloped. These findings provide important information for understanding the role of CNP/NPR-B signals on intestine with mesentery.

## Introduction

Natriuretic peptide receptor B (NPR-B) is known as a receptor for C-type natriuretic peptide (CNP), which contributes to the generation of intracellular second messenger cyclic guanosine-3ʹ,5ʹ-monophosphate (cGMP) by binding to CNP. The signal is involved in smooth muscle relaxation and blood pressure control and is recognized for its biological importance. Further, it has been predicted that CNP/NPR-B signaling has a different regulatory mechanism depending on the organ and size of a blood vessel [1, 2]. Recently, a human epidemiological study reported that CNP levels in the blood were high in hypertensive patients [3]. Whether this is due to a biological mechanism to high blood pressure or a result of a CNP-induced increase in blood pressure remains elusive. In addition, in the context of human obesity, it was reported that CNP is a suppressor of obesity as its level was decreased in the obese group [4–6]; however, another study showed no significant difference in blood CNP levels between the normal and obese groups [7]. Furthermore, CNP suppressed obesity in mice [8, 9] while CNP/NPR-B/cGMP promoted adipogenesis in an in vitro experimental system [10].

We have previously established a spontaneous mutant mouse strain as a short-limbed dwarfism (SLW) mouse. Mice homozygous for SLW (*Npr2*^*slw/slw*^) are defective in NPR-B function due to a frameshift mutation in *Npr2*, particularly in the exon-8 encoding the region present just under the transmembrane domain [11]. The phenotypes of *Npr2*^*slw/slw*^ include dwarfism [12], gastrointestinal (GI) disorders such as severe ileus-like condition with gas [11, 13, 14], erectile dysfunction in male reproductive organs [15] and significant reduction of white adipose tissue and triglyceride in the blood in adults [14]. We revealed that CNP relaxes the pyloric antrum and large intestine of normal mice but not *Npr2*^*slw/slw*^, demonstrating a site-specific direct effect of CNP on the GI tract [11]. Therefore, we previously believed that the GI disorder in *Npr2*^*slw/slw*^ arose from the direct effects of CNP/NPR-B signal deficiency on the GI tract. However, although the loss of NPR-B did not affect the levels of the ligand CNP, an electrolyte, and triglyceride in the blood in preweaning *Npr2*^*slw/slw*^, significant bloodless blood vessels and few adipocytes in the mesentery and abnormal intestinal development could also be factors for GI disorders [14]. These findings indicate that CNP/NPR-B signaling plays an essential role in regulating mesentery and/or intestinal blood flow.

However, it is unclear how CNP/NPR-B signal deficiency affects mesenteric vascular morphology. Many of the *Npr2*^*slw/slw*^ mice exhibited gastrointestinal disorders at 10 to 15 days of age. Therefore, in the present study, we analyzed the morphology of mesenteric vessels in *Npr2*^*slw/slw*^ mice, focusing on when they started to show their most distinctive phenotype with severe GI disorders in addition to survived adult *Npr2*^*slw/slw*^ mice.

## Main text

### Materials and methods

#### Mice

An *Npr2*^*slw/slw*^ strain was established from a mating between a founder male spontaneously mutant mouse in the ddY mouse colony at Okayama University and a C57BL/6J female [11, 12]. The mice in the mix background of ddY and C57BL/6J used in this study was described in the previous report [14]. The mice were maintained under standard 12 h light/dark conditions. Either heterozygotes or wild-type mice were used for comparison (referred to as controls); homozygotes from the same litter for controls were used and referred to as *Npr2*^*slw/slw*^. Mice were anesthetized by injecting a combined anesthetic containing 7.5% medetomidine hydrochloride, 8% midazolam, and 10% butorphanol tartrate in saline (10 µl/g) into the subcutaneous of the neck or intraperitoneal using a 29G syringe. Mice were euthanized by cut the diaphragm under anesthesia and immediately removed their whole gut.

All animal experiments were carried out in accordance with the institutional guidelines regarding animal care and handling, and the experimental protocol was approved by the Institutional Animal Care and Use Committee of the University of Tokyo.

#### Elastica van Gieson (EVG) staining

Whole GI tissue with mesentery was fixed in 10% buffered formalin at 4 °C overnight, dehydrated, and embedded in paraffin. Six-µm-thick sections were cut, placed on glass slides, and subjected to EVG and immunofluorescence staining.

For EVG staining, tissues were deparaffinized, hydrophilized, and immersed in 1% HCl in 70% ethanol for 3 min. The tissues were then immersed in resorcin-fuchsin solution (40321, Muto Pure Chemicals, Tokyo, Japan) for 2 h. Washing was performed with 100% ethanol for 3 min three times, followed by immersion in water, and treatment with iron-hematoxylin (40341 and 40351 Muto Pure Chemicals) for 15 min. After 30 min of rinsing under running water, the tissue was immersed in 5% Sirius Red (33061, Muto Pure Chemicals) in saturated picric acid for 15 min, quickly dehydrated, permeabilized, and embedded.

#### Immunofluorescence staining

Immunostaining was performed as previously described [14], and the primary antibodies used were rabbit polyclonal anti-alpha-smooth muscle actin (aSMA) antibody (ab5694, Abcam, Cambridge, UK, 1:100 dilution) and goat polyclonal lymphatic vessel endothelial receptor 1 (LYVE-1) antibody (AF2125, R&D systems, Minneapolis, MN, USA, 1:100 dilution). The secondary antibodies used were donkey anti-rabbit IgG H&L Alexa Fluor 488 (A21206, Thermo Fisher, Waltham, MA, USA, 1:1,000 dilution) and donkey anti-goat IgG H&L 594 (A11058, Thermo Fisher, 1:1,000 dilution).

#### Microscopy and acquisition

Stained images were acquired using a BZ-X710 all in one microscope (Keyence, Osaka, Japan) or an Olympus fluorescent microscope IX73 equipped with a color camera DP73 (Olympus, Tokyo, Japan) for P8 and P15 samples or adult samples, respectively. For the observation of P8 and P15 samples, autofocus imaging was done using a ×4 lens, and z-stacked images were generated with 10 section pictures with 0.5 µm intervals when using a ×40 lens. For the observation of adult samples, single focal images were observed using ×4, ×20, and ×40 lenses. Images were edited using Fiji software [16] and Photoshop (Adobe, San Jose, CA, USA). Mesenteric artery containing two layers of lamina elastica (LE) and parallelly running veins were selected for imaging.

#### Blood pressure measurement for adult mice

The blood pressure of mice was measured using a BP-98A blood pressure system (Softron, Tokyo, Japan) by the tail-cuff method. Systolic, diastolic, and heart rates were measured under a condition of non-anesthesia and retention using a mouse retention device. Measurements were obtained five times for each mouse, and their average values were shown in graphs.

#### Statistical analysis

Data are expressed as the mean ± standard deviation (SD). Dot plots were generated using GraphPad Prism7 software (GraphPad Software, San Diego, CA, USA). The area of the lumen of the blood vessel field was calculated using the Fiji software. The statistical significance of differences in mean values was assessed using the Mann–Whitney U test. There were no criteria used for including and excluding experimental units. Randomization was not used to allocate experimental units.

### Results

#### Vasodilation and undeveloped adipose cell in *Npr2*^*slw/slw*^

**Figure. 1:**
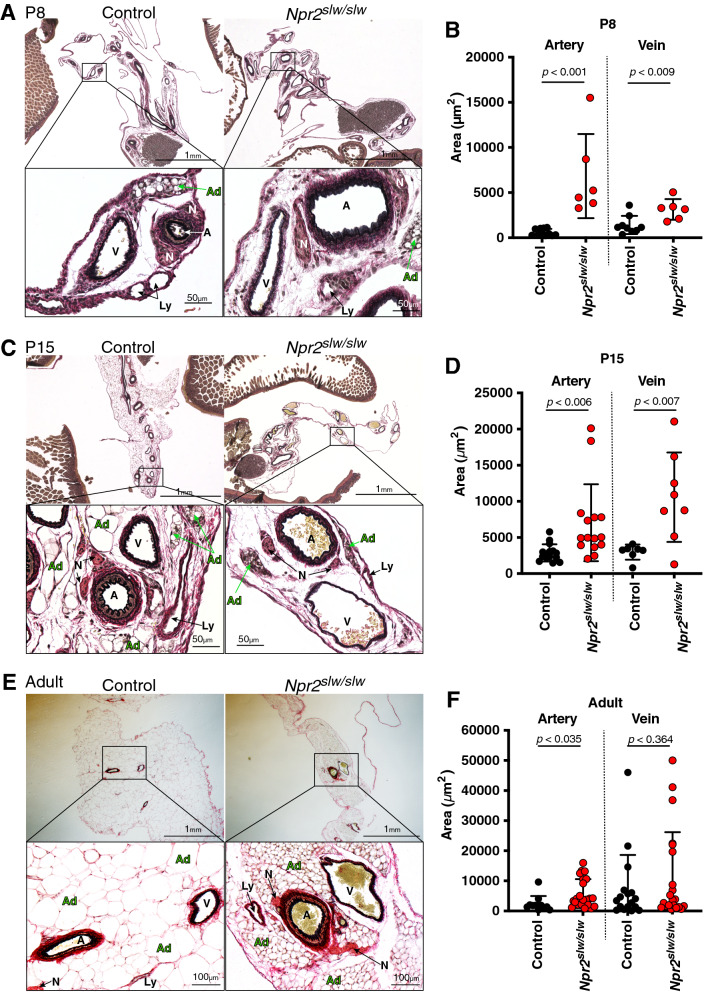
EVG staining and lumen area of the blood vessel. **A**, **C**, and **E**, EVG staining of the mesentery. *Npr2*^*slw/slw*^ (right) and litter control (left) at 1 week of age (P8) (**A**), 2 weeks of age (P15) (**C**), and 1 year of age (adult) (**E**). The bottom row shows a magnified image of the enclosure in the top row. A: artery, V: vein, Ad: adipocyte, Ly: lymph vessel. **B**, **D**, **and F**, Comparison of vascular lumen area in P8 (**B**), P15 (**D**), and adult (**F**). N = 3 mice for each genotype in P8 and P15. For adult samples, n = 4 and 6 for control and *Npr2*^*slw/slw*^ mice, respectively. The inner lines represent mean and SD, respectively. Dots represent the lumen size of each sample

In both control and *Npr2*^*slw/slw*^ mice, a bundle of mesenteric arteries, veins, lymph vessels, and nerves were arranged and surrounded by serosa. At 1 week of age (P8), a small adipose mass covered with a capsule was found in this bundle (Fig. 1A). At 2 weeks of age (P15), the inside of the mesentery was filled with further developed adipocytes in control, whereas no developed adipocytes were found in the *Npr2*^*slw/slw*^ mice (Fig. 1C).

The arteries in control at both P8 and P15 were covered with a developed adventitia, the inner and outer LE were contracted, and the blood vessels were thickened (Fig. 1A and C). In contrast, in *Npr2*^*slw/slw*^ mice at both ages, the LE was relaxed, and the blood vessels were dilated (Fig. 1A and C). The LE of veins was also contracted in control but was dilated in *Npr2*^*slw/slw*^ (Fig. 1A and C). At 1 year of age (adult), the LE of artery and vein was contracted in both control and *Npr2*^*slw/slw*^ (Fig. 1E). The inside of the mesentery was filled with large adipocytes in control, whereas small adipocytes were filled in the *Npr2*^*slw/slw*^ mice (Fig. 1E).

The lumen areas of arteries and veins were measured and compared between the control and *Npr2*^*slw/slw*^ mice, and the results showed that the areas were significantly larger for *Npr2*^*slw/slw*^ than for the control in P8 and P15 (Fig. 1B and D). In adult mice, the lumen area of arteries was larger than that of control mice, and there was no difference in the veins (Fig. 1F).

**Figure. 2.**
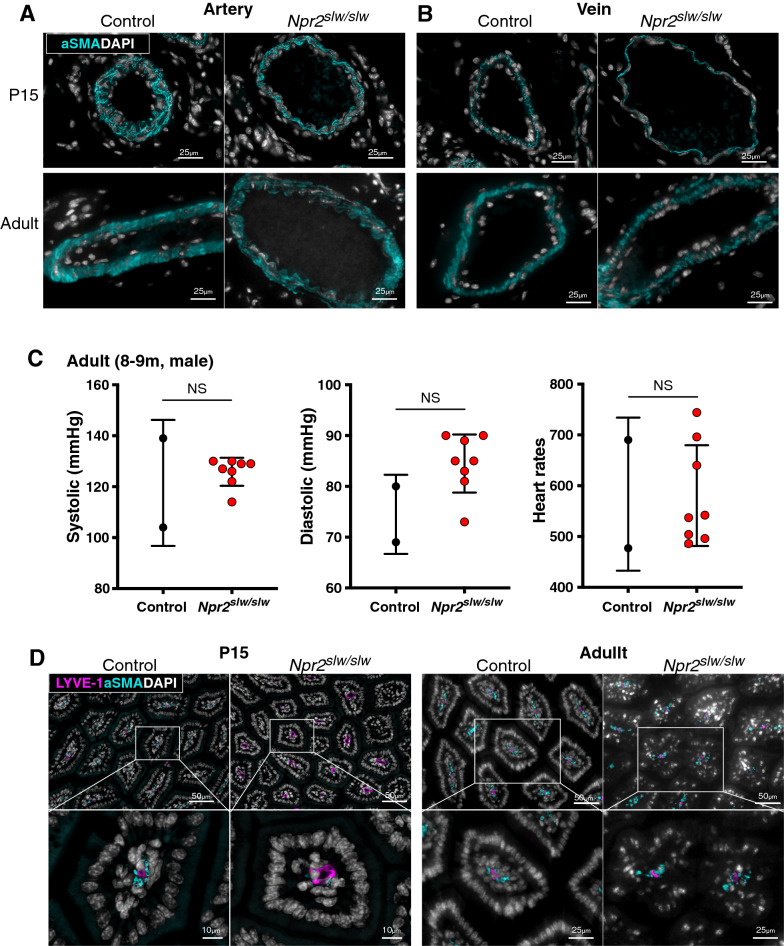
Cross-section and blood pressure. **A**, Artery of *Npr2*^*slw/slw*^ (right) and litter control (left) at P15 and adult. Cyan: smooth muscle, gray: nuclei. **B**, Veins of *Npr2*^*slw/slw*^ (right) and control (left) at P15 and adult. Cyan: smooth muscle, gray: nuclei. **C**, Blood pressure of adult male mice (8–9 months of age), n = 2 control and 8 *Npr2*^*slw/slw*^ mice, respectively. **D**, Cross-section of the villi. *Npr2*^*slw/slw*^ (right) and control (left) at P15 and adult. The bottom row shows magnified images of the enclosure in the top row. Cyan: smooth muscle, magenta: lacteal, and white: nucleus

In the arteries and veins of the smooth muscle, the smooth muscle in the control mice contracted in accordance with the LE, whereas that in *Npr2*^*slw/slw*^ was clearly elongated at P15 (Fig. 2A). However, smooth muscle in both control and *Npr2*^*slw/slw*^ adult mice contracted in accordance with the LE (Fig. 2B). Systolic, diastolic, and heart rates were similar between control and *Npr2*^*slw/slw*^ in adult male mice (Fig. 2C).

The villi of the entire intestine in control were developed uniformly, while *Npr2*^*slw/slw*^ exhibited uneven development at preweaning ages but normally developed at adult age [14]. The lacteals in the partially developed villi were dilated, and the smooth muscle in the villi was undeveloped in *Npr2*^*slw/slw*^ compared to that of the control in P15. At adult age, the lacteal and smooth muscle were recognized in villi of both control and *Npr2*^*slw/slw*^ mice, whereas the arrangement of nuclei of mucosal epithelial cell and lamina propria mucosae was disorganized in *Npr2*^*slw/slw*^ mice (Fig. 2D).

### Discussion

The CNP/NPR-B signal is known as an endothelium-derived vasorelaxant factor and has recently been recognized as an inhibitor of adipose hypertrophy. However, despite the lack of the CNP/NPR-B signal, *Npr2*^*slw/slw*^ unexpectedly displayed dilated blood vessels and undeveloped white adipose tissue in the mesentery. Several reasons may explain this. First, more CNP was available for binding to NPR-C, resulting in enhanced NPR-C function. NPR-C has a clearance of natriuretic peptides [17] while it also inhibits adenylyl cyclase via activation of phospholipase C by coupling with the Gi protein [18]. Indeed, NPR-C has been shown to have multiple functions: CNP/NPR-C is essential for vascular homeostasis and has a vasorelaxant effect [19, 20]. The lack of NPR-B may result in increased free CNP, which binds and activates NPR-C, leading to the dilation of mesenteric vessels and lacteals. Second, NPR-C has a binding affinity not only for CNP but also for other natriuretic peptides, such as atrial (ANP) and brain natriuretic peptide (BNP) [21]. As mentioned above, increased CNP binding to NPR-C may limit ANP and BNP binding to NPR-C, thus, enhancing the function of NPR-A, which is a receptor for ANP and BNP. In particular, ANP/NPR-A regulates blood pressure and fluid balance by relaxing major blood vessels [22–25]. This may have caused vasodilation in the *Npr2*^*slw/slw*^ mice. Given that relaxation of blood vessels by NPR-A or NPR-C is separable action from dilation, these are speculative but maybe probable. In addition, it was recently reported that NPR-A contributes to the reduction of white adipose tissue in humans and mice [26, 27]. Therefore, the suppression of adipogenesis in *Npr2*^*slw/slw*^ may also be due to the increased activity of NPR-A. Lastly, loss of CNP/NPR-B signal may cause the loss of its relaxing effect on the mesenteric vessels and become congested, giving the appearance of dilated vessels. Because experiments using mice have shown that CNP/NPR-B signals relax the mesenteric artery [28], it may be simply that the loss of NPR-B affected the mesenteric vessels. However, in adult *Npr2*^*slw/slw*^ mice, vasodilation was not evidently seen, and there appeared to be no abnormalities on blood pressure. Thus, it was suggested that CNP/NPR-B signal plays an essential role in the normal functions of blood vessels during pre-weaning ages and maintenance of peripheral tissues during adult age. Small adipocytes of adult *Npr2*^*slwslw*^ mice may have resulted from affected nutrient absorption owing to disorganized mucosal epithelial cells and lamina propria mucosae.

It remains to be answered whether the NPR-B of *Npr2*^*slw/slw*^, in which a premature stop codon eliminates the whole NPR-B structure under the transmembrane domain [11], contains only the extracellular ligand-binding domain that can bind to CNP but is nonfunctional or incapable of binding to CNP. That is, it remains to be determined whether the majority of CNP is cleared by clearance or CNP binds to NPR-C and enhances the NPR-C and/or NPR-A function. Further study would contribute to understanding the unique GI phenotype of *Npr2*^*slw/slw*^ mice. Model mice, such as *Npr2*^*slw/slw*^ mice, can be an essential source of information for understanding the function of genes and the effect of mutations. Therefore, the phenotype of the GI and vessel of *Npr2*^*slw/slw*^ mice would provide insight for the treatment of rare GI diseases, including the GI tract itself and secondary causes, and contribute to the elucidation of the CNP/NPR-B signaling mechanism in vivo.

## Limitation

Blood vessel samples from control and *Npr2*^*slw/slw*^ mice were observed and prepared in the same criteria, and the location in the mesentery was carefully determined according to the number of the LE and its morphology of the section. However, their location and distance may not be thoroughly the same. The number of control mice used for blood pressure was small (n = 2), limiting statistical significance. Therefore, no definite conclusion was not made regarding blood pressure of *Npr2*^*slw/slw*^ (n = 8).

## Data Availability

The dataset used and analyzed during the current study may be regarded with corresponding author on reasonable request.

## Abbreviations

NPR-B: Natriuretic peptide receptor B
CNP: C-type natriuretic peptide
GI: Gastrointestinal
ANP: Atrial natriuretic peptide
BNP: Brain natriuretic peptide
LE: Lamina elastica
